# Refractive and visual changes in the fellow eye after unilateral refractive surgery in patients with anisometropia

**DOI:** 10.3389/fmed.2026.1841715

**Published:** 2026-05-29

**Authors:** Yanjun Hu, Ruixuan Xu, Yuhua Tong, Meiting Yu

**Affiliations:** 1 Department of Ophthalmology, The Quzhou Affiliated Hospital of Wenzhou Medical University, Quzhou People's Hospital, Quzhou, China; 2 Clinical Medical College, Hainan Medical University, Haikou, China; 3 Department of Nursing, The Quzhou Affiliated Hospital of Wenzhou Medical University, Quzhou People’s Hospital, Quzhou, China

**Keywords:** anisometropia, fellow eye, LogMAR visual acuity, spherical equivalent, unilateral refractive surgery

## Abstract

**Objective:**

This study aimed to investigate the changes in visual acuity and refractive status of the fellow eye after unilateral refractive surgery in patients with anisometropia and to analyze their correlation with factors such as age, degree of anisometropia, and type of ametropia.

**Methods:**

A retrospective analysis was conducted on 43 patients with anisometropia who underwent unilateral refractive surgery at the Quzhou People’s Hospital, Zhejiang Province, from January 2023 to June 2025. All patients underwent femtosecond laser small incision lenticule extraction (SMILE) or femtosecond laser-assisted excimer laser *in situ* keratomileusis (FS-LASIK) on the eye with worse uncorrected visual acuity. Follow-up examinations of both eyes were performed preoperatively and at 1 day, 1 week, 1 month, 3 months, and 6 months postoperatively, assessing the uncorrected distance visual acuity and spherical equivalent (SE) of the fellow eye. Repeated-measures analysis of variance (ANOVA) was used to compare changes in the logarithm of the minimum angle of resolution (logMAR) visual acuity and SE of the fellow eye at different time points, and correlations with age, surgical eye SE, and preoperative anisometropia magnitude were analyzed.

**Results:**

The preoperative SE of the operated eye was −2.95 ± 1.84 D, and that of the fellow eye was (−0.25 ± 0.46) D, with a binocular anisometropia difference of 2.71 ± 1.69 D. Within 6 months postoperatively, the refraction of the operated eye remained stable (time main effect *p* = 0.263), recovering from a preoperative SE of −3.006 D to 0.038 D by 1 day postoperatively, with no significant differences between any postoperative time points (all *p* = 1.000). The logMAR visual acuity of the operated eye improved from 0.701 preoperatively to 0.017 at 1 day postoperatively and continued to optimize to −0.062 by 3 months postoperatively (superior to standard vision), with no significant fluctuations observed between postoperative time points (*p* = 0.340). In contrast, the refraction of the fellow eye showed significant changes over time (*p* = 0.050), with a transient myopic shift at 1 week postoperatively (−0.573 D), which recovered to −0.134 D by 6 months postoperatively, showing no significant difference from preoperative values (*p* = 1.000). The logMAR visual acuity of the fellow eye remained stable at all time points (*p* = 0.223), consistently remaining near zero. Age, gender, and other covariates had no significant effect on the change trends of either eye (all *p* > 0.05).

**Conclusion:**

In patients with anisometropia undergoing unilateral refractive surgery, the fellow eye may experience transient, reversible refractive fluctuations in the early postoperative period, most pronounced at 1 week postoperatively. However, logMAR visual acuity remains stable, indicating the good safety of this surgical approach. Clinicians need not intervene excessively or perform prophylactic bilateral surgery out of concern for visual acuity decline in the fellow eye.

## 1 Introduction

Anisometropia is a common type of ametropia in clinical practice, with a prevalence of approximately 2.7–20.0% due to differences in spherical equivalent (1, 2). The prevalence of non-amblyopic anisometropia increases systematically with age, with linear regression showing a 1% increase every 7 years (3, 4). Recent studies have found that anisometropia and anisomyopia are associated with systemic parameters such as age, grade level, parental education level, and lifestyle factors (such as time spent on indoor reading and writing) (5, 6). In contrast, anisohyperopia and anisoastigmatism are not related to lifestyle factors. Anisometropia is primarily associated with the interocular difference in axial length, suggesting that its underlying mechanism is primarily related to changes in the posterior segment of the eye. Anisometropia significantly causes changes in contrast sensitivity, visual evoked potentials, peripapillary retinal nerve fiber layer thickness, and optic disc area (7–9). Moreover, childhood anisometropia is more likely to lead to strabismus and amblyopia (10). Anisometropia may lead to binocular visual dysfunction, including decreased accommodation and convergence, thereby exacerbating visual fatigue (11). Binocular function decreases with the increasing degree of anisometropia (12). Myopic anisometropia of more than 2D can cause a significant binocular vision impairment (13). All types of anisometropia impair binocular fusion and stereopsis to varying degrees, with hyperopic anisometropia having the most significant impact, particularly on near stereopsis (14). Spectacle correction is the most common method for correcting refractive errors. However, when the refractive difference between the two eyes exceeds 2.50 D, wearing spectacles for correction often leads to fusion difficulties, as anisometropia results in aniseikonia and prismatic effects, affecting spectacle tolerance (15). Personalized refractive correction can improve visual discomfort and enhance visual quality (16). Surgical treatment for anisometropia is predictable, effective, and safe (17) and can also improve patients’ uncorrected visual acuity, reduce anisometropia, maintain or improve postoperative best corrected visual acuity, and simultaneously eliminate aniseikonia caused by wearing spectacles. Furthermore, the surgical treatments may prevent or improve complications associated with anisometropia, such as fusion dysfunction, loss of stereopsis, unilateral amblyopia, and strabismus, promoting the recovery of binocular single vision (18). However, during routine clinical practice, the authors observed that some patients with anisometropia who underwent unilateral refractive surgery experienced a decline in fellow-eye visual acuity over time. This raises the following questions, including: (i) Do all patients with anisometropia undergoing unilateral refractive surgery experience a decline in fellow-eye visual acuity? (ii) If so, how long after surgery does the visual acuity decline begin? What is the progression of the visual acuity decline? Does it continuously decline, and when does it peak? Or does it decline to a certain level and then recover? (iii) If a decline does occur, are interventions effective? What type of intervention works best? When should intervention be applied? (iv) To avoid the impact of visual acuity decline on military enrollment or further education, what preventive strategies should be adopted? Should bilateral surgery be chosen directly? To explore these questions, this study aimed to investigate the changes in visual acuity and refractive status before and after unilateral refractive surgery in anisometropic patients.

## 2 Materials and methods

### 2.1 General information

Forty-three patients who underwent unilateral refractive surgery at the Quzhou People’s Hospital, Zhejiang Province, from January 2023 to June 2025 were selected. Among them, 83.72% (36 patients) were men, and 16.28% (7 patients) were women, and the age range of the participants was 17 to 32 years, with a mean age of 19.70 ± 3.82 years. All patients underwent unilateral corneal refractive surgery on the eye with worse uncorrected visual acuity. Surgical methods included femtosecond laser small incision lenticule extraction (SMILE) and femtosecond laser-assisted excimer laser *in situ* keratomileusis (FS-LASIK). The refractive status of the surgical eye was essentially stable, with an annual increase in refractive power not exceeding 0.50 D in the 2 years preoperatively. Patients discontinued soft contact lens wear for at least 1 week, rigid contact lenses for at least 3 weeks, and orthokeratology lenses for at least 3 months before undergoing preoperative ophthalmic examinations, including uncorrected distance visual acuity, slit-lamp microscopy, intraocular pressure measurement, axial length measurement, corneal topography, manifest refraction, cycloplegic autorefraction, and ultra-widefield Optos fundus imaging. Other ocular diseases and systemic conditions were excluded, such as active ocular inflammation, glaucoma, uveitis, significant media opacities affecting vision, retinal detachment, and immune diseases. Patients were fully informed of the treatment plan, precautions, and possible postoperative conditions before surgery. Patients fully understood the surgery, accepted the associated risks, agreed to and could tolerate the surgery, and signed the informed consent form.

### 2.2 Methods

All patients completed detailed ophthalmic examinations preoperatively, and all surgeries were performed by experienced surgeons. Among them, 40 patients chose SMILE, and 3 patients chose FS-LASIK. Preoperatively, routine conjunctival sac irrigation and periorbital disinfection were performed, followed by topical anesthesia with two instillations of Proparacaine Hydrochloride Eye Drops. The SMILE surgical method used VisuMax 3.0 (Carl Zeiss, Germany), with a pulse frequency of 500 kHz and pulse energy of 125 nJ. Corneal cap thickness was designed to be 100–125 μm, with a treatment zone diameter of 6.2–7.0 mm. The FS-LASIK surgical method used VisuMax 3.0 + MEL90 (Carl Zeiss, Germany), with a corneal flap thickness of 95–100 μm and a treatment zone diameter of 6.3–6.8 mm. Tobramycin dexamethasone eye drops were instilled at the end of both procedures. For both groups, beginning on day 1 postoperatively, levofloxacin 0.5% ophthalmic solution (Cravit®, Santen Pharmaceutical Co., Ltd., Osaka, Japan) were instilled four times/day for 1 week; fluorometholone 0.1% ophthalmic suspension (FML®, AbbVie Ltd., Mayo, Ireland) was instilled four times/day, tapered weekly, and discontinued after 1 month; sodium hyaluronate 0.3% preservative-free unit-dose eye drops (Hyaluronic Acid, Essex Bio-Technology Ltd., Zhuhai, China) was instilled four times/day; from postoperative day 3, esculin and digitalisglycosides eye drops (Stulln®, Pharma Stulln GmbH, Stulln, Germany) were instilled three times/day.

### 2.3 Postoperative follow-up

All surgeries were completed successfully. Follow-up examinations were performed at 1 day, 1 week, 1 month, 3 months, and 6 months postoperatively. Outcomes included uncorrected distance visual acuity, intraocular pressure, and refraction (spherical equivalent from manifest refraction) of both eyes. Uncorrected distance visual acuity was measured using the standard logarithmic visual acuity chart, and the values were recorded in logMAR units. The standard logarithmic visual acuity chart (developed by Wenzhou Medical University) was used, employing the early treatment diabetic retinopathy study (ETDRS) layout format, with 200 Lux (using a standard visual acuity chart light box) and testing distance of 4 m. Visual acuity values were recorded in logMAR units. Guessing was permitted, with termination defined as three incorrect recognitions out of five optotypes on the same line.

### 2.4 Statistical analysis

This retrospective study used SPSS 26.0 statistical software for data analysis. Based on the interval-scale measurement properties and higher sensitivity in statistical analyses, the logMAR visual acuity was selected as the primary visual outcome (19, 20). Comparisons of visual acuity and refractive status before and after surgery were performed using repeated-measures analysis of variance (ANOVA). A *p*-value of <0.05 was considered statistically significant.

## 3 Results

The preoperative spherical equivalent (SE) of the operated eye was −2.95 ± 1.84 D, and that of the fellow eye was −0.25 ± 0.46 D, yielding a preoperative anisometropia difference of 2.71 ± 1.69 D. Repeated-measures analysis of covariance was performed, with age, SE of the operated eye, preoperative magnitude of anisometropia, and baseline SE of the fellow eye included as covariates, to examine the effect of time (preoperatively, 1 day, 1 week, 1 month, 3 months, and 6 months postoperatively) on the SE and logMAR visual acuity of both the operated and fellow eyes. The estimated marginal means for SE and logMAR of the operated and fellow eyes at each time point are shown in Table 1.

**Table 1:** Estimated marginal means of binocular spherical equivalent (D) and LogMAR visual acuity at different time points (Mean ± Standard error).

Time	Surgical eye		Fellow eye	
	SE(D)	LogMAR	SE(D)	LogMAR
Preop	−3.006 ± 0.000	0.701 ± 0.049	−0.246 ± 0.000	0.006 ± 0.019
Day1	0.038 ± 0.095	0.017 ± 0.026	−0.307 ± 0.085	−0.008 ± 0.026
Week1	0.120 ± 0.067	−0.017 ± 0.018	−0.573 ± 0.098	0.052 ± 0.031
Month1	0.071 ± 0.074	−0.041 ± 0.014	−0.334 ± 0.106	0.039 ± 0.035
Month3	0.030 ± 0.083	−0.062 ± 0.014	−0.237 ± 0.100	−0.009 ± 0.024
Month6	0.037 ± 0.080	−0.046 ± 0.012	−0.134 ± 0.089	−0.004 ± 0.023

### 3.1 Changes in spherical equivalent

**Figure 1:**
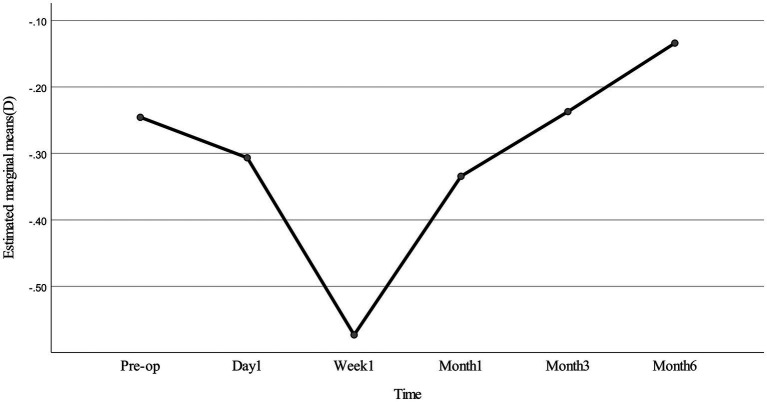
Estimated marginal means for SE of the fellow eye. Covariates in the model were set to the following values: Age = 19.70 years old, SE of Surgical eye = −2.9535D, degree of anisometropia = −2.7078D, the fellow eye preoperative SE baseline = −0.2456D. Pre-op indicates preoperative, DAY1 indicates 1 day postoperatively, week1 indicates 1 week postoperatively, Month1 indicates 1 month postoperatively, Month3 indicates 3 month postoperatively, Month6 indicates 6 month postoperatively.

The results of repeated-measures ANOVA showed significant differences in the SE of the fellow eye at different time points [multivariate tests: Wilks Lambda = 0.571, *F* (10, 21) = 2.176, *p* = 0.050, partial η^2^ = 0.429], indicating that the refraction of the fellow eye experienced significant changes over time during the 6-month postoperative period. The Mauchly’s test of sphericity was significant (Mauchly *W* = 0.369, *p* = 0.1); therefore, the Greenhouse–Geisser correction was applied. The corrected univariate test showed that the main effect of time was not statistically significant [*F*(3.829, 145.517) = 1.953, *p* = 0.108, partial η^2^ = 0.049], suggesting that the time effect was primarily dependent on the multivariate test results. Trend analysis revealed a significant quintic trend in the SE changes [*F* (1, 22)= 8.260, *p* = 0.007, partial η^2^ = 0.179], indicating a fluctuating postoperative pattern characterized by “an initial increase in the early postoperative period, followed by gradual recovery.” Estimated marginal means showed that the SE of the fellow eye peaked at −0.573 D at 1 week postoperatively and recovered gradually to −0.134 D by 6 months after surgery (Figure 1).

**Figure 2:**
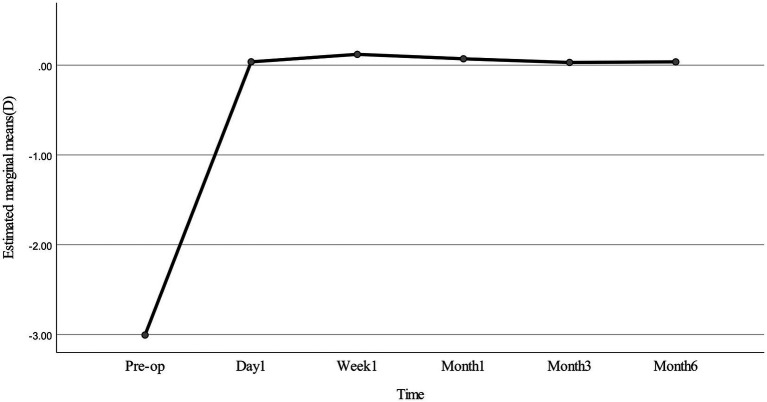
Estimated marginal means for SE of the operated eye. Covariates in the model were set to the following values: Age = 19.70 years old, SE of Surgical eye = −2.9535D, degree of anisometropia = −2.7078D, the fellow eye preoperative SE baseline = −0.2456D. Pre-op indicates preoperative, DAY1 indicates 1 day postoperatively, week1 indicates 1 week postoperatively, Month1 indicates 1 month postoperatively, Month3 indicates 3 month postoperatively, Month6 indicates 6 month postoperatively.

No significant time main effect was observed for the refractive power of the operated eye at any postoperative time point [multivariate test: Wilks’ Lambda = 0.669, *F* (10, 23) = 1.335, *p* = 0.263, partial η^2^ = 0.331], indicating stability throughout the 6-month postoperative period. The trend analysis revealed that none of the polynomial trends (linear, quadratic, cubic, quartic, or quintic) reached statistical significance (all *p* > 0.05), further confirming refractive stability. Estimated marginal means showed that the preoperative SE of the operated eye was −3.006 D (myopic state), which shifted to 0.038 D (emmetropic state) by 1 day postoperatively. Pairwise comparisons with Bonferroni correction demonstrated that the differences in SE between the preoperative time point and each postoperative time point were highly significant (all *p* < 0.001); however, no significant differences were found between any of the postoperative time points (all *p* = 1.000; Figure 2).

### 3.2 Visual acuity change

**Figure 3:**
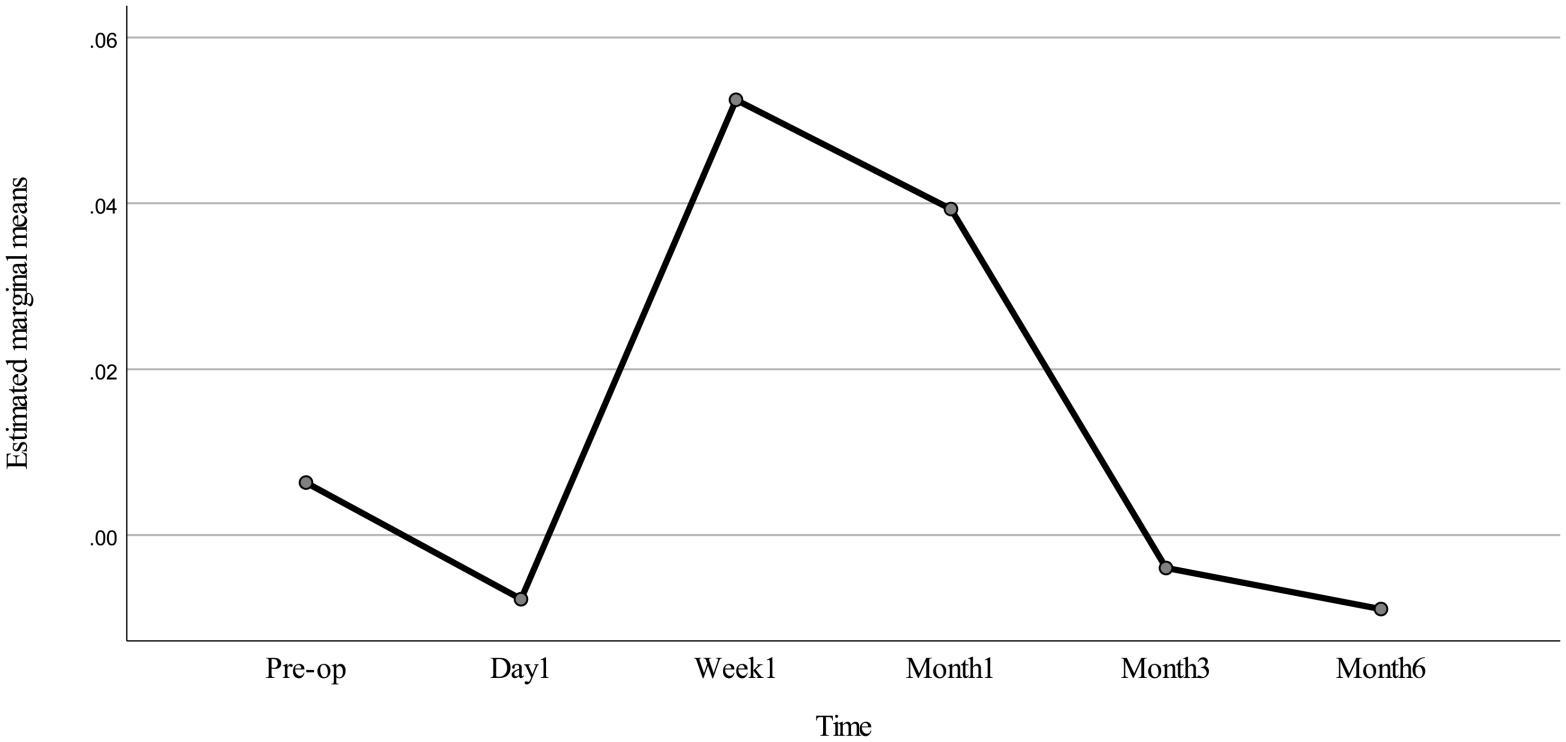
Estimated marginal means for VA of the fellow eye. Covariates in the model were set to the following values: Age = 19.70 years old, SE of Surgical eye = −2.9535D, degree of anisometropia = −2.7078D, the fellow eye preoperative SE baseline = −0.2456D. Pre-op indicates preoperative, DAY1 indicates 1 day postoperatively, week1 indicates 1 week postoperatively, Month1 indicates 1 month postoperatively, Month3 indicates 3 month postoperatively, Month6 indicates 6 month postoperatively.

Changes in logMAR visual acuity of the fellow eye did not reach statistical significance. Multivariate tests showed no significant time main effect [Wilks Lambda = 0.931, *F*(10, 378) = 1.378, *p* = 0.189], and the univariate tests also showed no significant time main effect [Greenhouse–Geisser correction: *F*(3.146, 119.539) = 1.475, *p* = 0.223, partial η^2^ = 0.037]. Trend analysis revealed a significant quintic trend for visual acuity change [*F* (1, 22)= 10.941, *p* = 0.002, partial η^2^ = 0.224], indicating minor fluctuations in visual acuity, although this trend did not reach statistical significance in the overall test (Figure 3).

**Figure 4:**
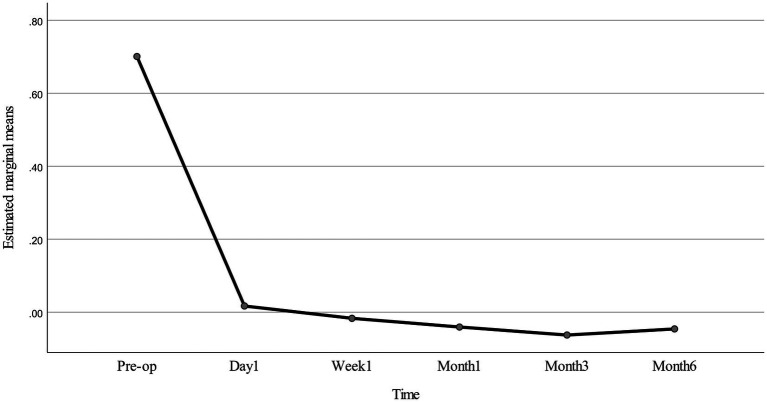
Estimated marginal means for VA of the operated eye. Covariates in the model were set to the following values: Age = 19.70 years old, SE of Surgical eye = −2.9535D, degree of anisometropia = −2.7078D, the fellow eye preoperative SE baseline = −0.2456D. Pre-op indicates preoperative, DAY1 indicates 1 day postoperatively, week1 indicates 1 week postoperatively, Month1 indicates 1 month postoperatively, Month3 indicates 3 month postoperatively, Month6 indicates 6 month postoperatively.

The multivariate analyses for the operated eye showed no significant time main effect [Wilks’ Lambda = 0.932, *F*(10, 358) = 1.274, *p* = 0.244]. Furthermore, the Mauchly’s test of sphericity was significant (Mauchly’s *W* = 0.003, *p* < 0.001), indicating violation of the sphericity assumption; therefore, the Greenhouse–Geisser correction was applied. The corrected univariate analysis revealed that the main effect of time did not reach statistical significance [*F*(1.705, 61.383) = 1.072, *p* = 0.340, partial η^2^ = 0.029], indicating stability of visual acuity across postoperative time points. A trend analysis revealed no significant polynomial trends (all *p* > 0.05), confirming the stability of visual acuity. Pairwise comparisons with Bonferroni correction demonstrated highly significant differences in visual acuity between the preoperative and postoperative time points (Figure 4).

### 3.3 Influence of other variables

Age had no significant impact on the change trend of the fellow eye [Time × Age interaction: Wilks Lambda = 0.648, *F* (10, 21) = 1.572, *p* = 0.165, partial η^2^ = 0.352]. Similarly, no significant impact on the change trend of the operated eye (Time × Age interaction: Wilks’ Lambda = 0.763, *F* (10, 23) = 0.840, *p* = 0.596, partial η^2^ = 0.237) was observed.

Surgical eye SE, degree of anisometropia, and preoperative SE baseline showed no significant interaction with the change trend of the fellow-eye changes (*p*-values could not be calculated for all due to degrees of freedom issues, but partial η^2^ were 0.000), suggesting negligible effects.

This study did not detect significant effects of age or gender on the trajectories of postoperative refractive or visual acuity changes.

## 4 Discussion

This study, through a 6-month follow-up of 43 patients undergoing unilateral refractive surgery, found that the operated eye was successfully corrected from myopia to emmetropia, with the primary efficacy endpoint essentially achieved by 1 day postoperatively and stably maintained throughout the follow-up period. In contrast, the fellow eye exhibited significant temporary myopic shift at 1 week postoperatively, which resolved by 6 months. Notably, throughout this process, the logMAR visual acuity of the fellow eye remained stable without significant fluctuations. The waveform trend observed in this study (“deepening at 1 week, recovery by 6 months,” with a significant quintic trend) further confirms the temporary and reversible nature of the change. The phenomenon of increased myopia in the fellow eye during the early postoperative period may stem from the reconstruction process of binocular visual balance. In general, anisometropic patients inherently have a certain degree of inequality in accommodative ability between the two eyes, resulting in a state of interocular accommodative difference (24–26). Corneal refractive surgery for myopia corrects refraction by altering the curvature of the anterior corneal surface, effectively transferring the power of the corrective lens to the cornea. With the elimination of the vertex distance between the lens and the cornea, the accommodative demand for near work increases (27). The visual fluctuation in the fellow eye during the early postoperative period is speculated to be related to decreased accommodative function. Accommodation typically decreases at 1 week after SMILE or FS-LASIK, begins recovery after 1 month, and exceeds preoperative levels by 3 months (23, 28, 29). The reasons are mild corneal edema 1 week postoperatively, decreased regularity and symmetry of the anterior surface, resulting in decreased contrast sensitivity, increased higher-order aberrations, and a postoperative delay in objective visual quality (21, 30, 31). The fovea cannot obtain a clear accommodative stimulus, and the amplitude of accommodation (AMP) and accommodative facility (AF) temporarily decrease (32–35). The early postoperative hyperopic shift also significantly increases accommodative demand for near work (36). As accommodative demand increases, accommodative response correspondingly decreases, resulting in accommodative lag. Accommodative lag means the eye’s focus falls behind the retina, leading to persistent retinal blur. To maintain a relatively clear image, the visual system must continuously make fine adjustments, leading to image instability (37). This change typically peaks around 1 week postoperatively and then gradually resolves as the central nervous system adapts (38). This study involved only unilateral surgery. A significant accommodative lag in one eye, with persistent blurry imaging (due to accommodative lag) (22), may disrupt binocular fusion. The brain may temporarily suppress the blurry image and rely more on the contralateral eye with clear imaging, triggering the mismatch and disorder of binocular accommodation (39). After the unilateral surgery, the abrupt change in the refractive state of the surgical eye (from high myopia to emmetropia) might induce transient accommodative spasm in the fellow eye to cope with the new visual demands (40).

The finding is noteworthy that, although refraction changed significantly, logMAR visual acuity, reflecting visual function, was not impaired, which may be because logMAR visual acuity is insensitive to minor refractive changes (<0.5 D) within the normal range, and young patients (mean age = 19.7 years) have strong accommodative compensation ability. Although not statistically significant, minor physiological fluctuations may be observed from the estimated marginal means (Table 1), showing a slight decrease in visual acuity from 1 week to 1 month postoperatively compared with preoperatively. Clinically, a few patients indeed experience decreased visual acuity in the fellow eye during recovery. For example, patient No. 37, with a preoperative right eye −1.50 DS/−0.50 DC and left eye −0.50 DS, had preoperative uncorrected logMAR visual acuity of 0.5 (Snellen fraction 20/63) in the right eye and 0 (Snellen fraction 20/20) in the left eye, and had noticed decreased vision in the right eye for 3 years and usually did not wear glasses. Due to plans to apply for military/police academies, refractive surgery was chosen. After complete preoperative evaluation, SMILE was performed on the right eye. At the 1 week postoperative follow-up, the patient complained of decreased vision in the left eye. Examination revealed uncorrected visual acuity: right eye −0.08 (Snellen fraction 20/16) and left eye 0.15 (Snellen fraction 20/30). Manifest refraction showed no improvement with correction in the right eye, whereas the left eye achieved a corrected visual acuity of −0.08 (Snellen fraction 20/16) corrected with −1.00 DS. The patient was instructed to continue 0.1% Fluorometholone eye drops three times/day, tapered weekly, and discontinued after 3 weeks; sodium hyaluronate eye drops four times/day; and esculin and digitalisglycosides eye drops three times/day, and to begin accommodation training with ±2.00 D flippers for 15 min/day. At 1 month postoperatively, the patient’s uncorrected visual acuity of the right eye was −0.08 (Snellen fraction 20/16) and that of the left eye was 0 (Snellen fraction 20/20). Manifest refraction showed no improvement with correction in the right eye, while the left eye achieved a corrected visual acuity of −0.08 (Snellen fraction 20/16) with −0.25 DS/−0.50 DC. At 3 months postoperatively, uncorrected visual acuity in the right eye was −0.18 (Snellen fraction 20/13) and in the left eye was 0 (Snellen fraction20/20). Manifest refraction showed no improvement with correction in the right eye, while the left eye achieved a corrected visual acuity of −0.08 (Snellen fraction 20/16) with −0.25 DS/−0.75 DC. The patient reported discontinuing flipper exercises during the past 1 month postoperatively because of academic pressure. The patient was instructed to continue sodium hyaluronate eye drops four times/day, and flipper exercises were suggested to continue. During subsequent follow-up, the patient’s left eye visual acuity remained stable at 0 (Snellen fraction 20/20). These observations led to the conclusion that postoperative refractive and visual fluctuations in the fellow eye are likely a functional adaptive process rather than an organic pathological change. This understanding may facilitate more accurate postoperative patient education, alleviates anxiety, and suggests that appropriate visual function training during follow-up may help boost patient confidence.

In this study, age had no significant effect on the fellow eye. However, as the observed subjects were primarily young people (mean age = 19.70 years, maximum age = 32 years) with generally strong accommodation, the results do not apply to individuals aged ≥35 years with presbyopia or pre-presbyopia (41, 42). Additionally, the gender sample disparity (36 men and 7 women) may lead to insufficient statistical power to detect true gender differences.

This study employed a doubly multivariate repeated-measures ANOVA to analyze two related indicators simultaneously. The study also included age, surgical eye refraction, and anisometropia magnitude as covariates to control for confounding factors. Complete data from preoperative to 6 months postoperative were collected for all observed subjects, allowing the observation of the complete change trajectory. However, this study has some limitations. First, the sample size was limited (43 cases), with a notable gender imbalance (men:women = 36:7, 83.7% men). This imbalance reflects the clinical reality that young male patients undergoing unilateral refractive surgery for military recruitment purposes constitute the majority of this cohort. Consequently, subgroup analyses (such as gender differences) lack sufficient statistical power (estimated at only 25–40% to detect interaction effects), and the data still showed some fluctuation. Therefore, further in-depth research with larger, more balanced samples is required to validate the exploratory findings regarding gender effects. Although the main effect of time on SE was marginally significant (*p* = 0.050), the effect size (η^2^*p* = 0.429) was large, and the observed power was approximately 0.75, suggesting that the conclusion is relatively reliable. Second, the follow-up period in this study was limited to 6 months postoperatively. Therefore, it is not possible to determine whether late-onset refractive changes or long-term regression occur at or beyond 1 year after surgery. Future studies should extend the follow-up duration to at least 1–2 years to more comprehensively assess the long-term prognosis. Third, accommodative parameters (such as accommodative amplitude, accommodative lag, and accommodative facility) were not directly measured in this study. Consequently, the mechanistic hypothesis regarding “accommodative spasm” requires further validation. Future studies should combine objective accommodative function testing (especially using open-field autorefractors or infrared optometers to measure dynamic accommodative responses) to directly validate this hypothesis. All participants in this study were young adults (aged 17–32 years). Therefore, the conclusions of this study cannot be generalized to the pre-presbyopic (≥35 years) or presbyopic population.

## 5 Conclusion

This study showed that not all anisometropic patients undergoing unilateral refractive surgery experience decreased visual acuity in the fellow eye. Although SE shows transient early postoperative fluctuations, logMAR visual acuity is not significantly affected within the first 6 months postoperatively, suggesting that this surgical approach has good safety and stability in visual quality recovery. These findings also suggest that clinicians need not overintervene even when objective examinations reveal refractive fluctuations in the fellow eye. This surgical approach may reduce the economic burden of myopia treatment (43). Furthermore, clinicians should provide clear explanations to alleviate patient anxiety, informing them that subjective visual quality typically does not deteriorate. Therefore, this study does not recommend prophylactic refractive correction surgery on the fellow eye solely due to concerns about postoperative visual acuity decline.

## Data Availability

The original contributions presented in the study are included in the article/Supplementary material, further inquiries can be directed to the corresponding author.
